# Sarcopenia Prevalence among Hospitalized Patients with Severe Obesity: An Observational Study

**DOI:** 10.3390/jcm13102880

**Published:** 2024-05-13

**Authors:** Raffaella Cancello, Ettore Brenna, Davide Soranna, Antonella Zambon, Valentina Villa, Gianluca Castelnuovo, Lorenzo Maria Donini, Luca Busetto, Paolo Capodaglio, Amelia Brunani

**Affiliations:** 1 Obesity Unit, Laboratory of Nutrition and Obesity Research, Department of Endocrine and Metabolic Diseases, IRCCS Istituto Auxologico Italiano, 20121 Milan, Italy; r.cancello@auxologico.it; 2 Biostatistic Unit, IRCCS Istituto Auxologico Italiano, 20121 Milan, Italy; e.brenna@auxologico.it (E.B.); d.soranna@auxologico.it (D.S.); antonella.zambon@unimib.it (A.Z.); 3 Department of Statistics and Quantitative Methods, University of Milan-Bicocca, 20126 Milan, Italy; 4 Psychology Research Laboratory, IRCCS Istituto Auxologico Italiano, 20824 Piancavallo-Verbania, Italy; v.villa@auxologico.it (V.V.); gianluca.castelnuovo@unicatt.it (G.C.); 5 Department of Psychology, Catholic University of Milan, 20123 Milan, Italy; 6 Department of Experimental Medicine, Sapienza University, 00185 Rome, Italy; lorenzomaria.donini@uniroma.it; 7 Department of Medicine, University of Padova, 35122 Padova, Italy; luca.busetto@unipd.it; 8 Department of Surgical Sciences, Physical Medicine and Rehabilitation, University of Turin, 10126 Turin, Italy; p.capodaglio@auxologico.it; 9 Laboratory of Biomechanics, Rehabilitation and Ergonomics, IRCCS Istituto Auxologico Italiano, 28824 Piancavallo-Verbania, Italy

**Keywords:** obesity, sarcopenia, sarcopenic obesity, handgrip, skeletal muscle mass, quality of life

## Abstract

**Background**: Sarcopenic obesity (SO) is the combination of excess fat, skeletal muscle and muscular strength/function deficit. The ESPEN/EASO have proposed new diagnostic criteria, but the SO prevalence in patients with severe obesity remains to be established. The aim of this study was to establish the SO prevalence in a large cohort of inpatients with obesity, considering sex, age, BMI, type, and number of concomitant diseases. **Methods**: Patient data of both genders aged between 18 and 90 years with a body mass index (BMI) of ≥30 kg/m^2^ underwent hospital evaluation including bioelectrical impedance analysis (BIA) and handgrip strength (HS). QoL scores were obtained by the Psychological General Well-Being Index questionnaire. The study was approved by the institutional Ethic Committee. **Results**: Among the 3858 patients, 444 (11.51%) exhibited a strength deficit, while 3847 (99.71%) had skeletal muscle mass deficit. The prevalence of SO was then 11.48%, with higher rates in women (12.39%), in individuals aged >70 years (27%), and in those reporting a ‘poor’ QoL (12.6%). No significant difference in SO prevalence was found when stratifying by BMI (30–40 kg/m^2^ vs. >40 kg/m^2^, *p* = 0.1710). In SO patients, osteoarticular diseases (57%), hypertension/heart failure (38%), type 2 diabetes mellitus (34%), and obstructive sleep apnea (32%) were the more frequent comorbidities. **Conclusions**: The application of ESPEN/EASO-SO criteria in a cohort of inpatients with severe obesity revealed 11.48% SO prevalence, which was associated with age (particularly > 70 years), gender (women), but not BMI, as determinants. Disease staging and QoL screening may improve the identification of SO high-risk patients.

## 1. Introduction

Sarcopenia, defined by the European Working Group on Sarcopenia in Older People (EWGSOP) as the simultaneous loss of skeletal muscle mass and reduction in muscular function, is a common condition among the elderly, contributing to a heightened risk of falls, fractures, motor disability, and increased mortality [1,2].

Sarcopenic obesity (SO), characterized by an excess of adiposity alongside diminished skeletal muscle mass and/or function, is now acknowledged as a clinical concern [3]. Despite numerous studies investigating SO prevalence, the estimates vary significantly, with reported rates ranging from 0% to over 41% [3]. Factors influencing SO prevalence include the muscle mass measurement alone for sarcopenia diagnosis, the use of dual-energy X-ray absorptiometry (DXA) over bioelectrical impedance analysis (BIA) for muscle mass assessment, and the focus on individuals aged 75 years and older [2,3]. 

Indeed, SO serves as a prognostic indicator for disability and survival, increasing the risk of obesity- and age-related diseases. Excess adiposity and fat redistribution are linked to systemic inflammation and fat infiltration in skeletal muscle, exacerbating mitochondrial dysfunction and inflammatory cytokine production, worsening complications in patient with obesity [3,4,5,6]. The challenge in defining SO lies in the variety of methods used to define obesity and assess muscle mass and strength, leading to a lack of universally recognized diagnostic criteria [4,5,6,7,8]. 

Recently, an international expert panel met and discussed a Consensus Statement for SO definition and diagnostic criteria; for the relevance of the results, both shared by the European Society for Clinical Nutrition and Metabolism (ESPEN) and the European Association for the Study of Obesity (EASO), two articles were published [9,10]. A recommendation basis was created for a worldwide plan that included SO definition, screening, diagnosis, and staging using a decision algorithm to direct the diagnostic procedure that includes: (1) the screening of patients by high BMI or elevated waist circumference and surrogate parameters for sarcopenia (symptoms, clinical suspicion, and/or questionnaires); (2) diagnosis of patients by testing muscle function followed by body composition analysis; and (3) staging, if positive for sarcopenic obesity, based upon the absence (stage I) or presence (stage II) of attributable clinical complications such as functional disabilities, cardiovascular, and/or respiratory diseases [9,10].

Since ESPEN and EASO experts advocate that the proposed SO definition and diagnostic criteria may be implemented in clinical practice, thus encouraging the application, we then decided to estimate the SO prevalence by applying the consensus criteria in a large cohort of hospitalized patients suffering from severe obesity that considered the prevalence by sex, age, BMI, type, and number of concomitant diseases. 

In addition, with the intent to expand on the applicability of ESPEN/EASO-SO diagnostic criteria and better characterize SO patients, the level of quality of life (QoL) was screened with the Psychological General Well-Being Index (PGWBI) questionnaire. 

## 2. Materials and Methods

### 2.1. Patients

Inpatients with severe obesity consecutively admitted from April 2018 to December 2021 to the San Giuseppe Hospital, IRCCS Istituto Auxologico Italiano, Piancavallo (Verbania, Italy) for a 4-week multidisciplinary obesity rehabilitation program (i.e., metabolic, nutritional, and psychological rehabilitation) were considered. Data of patients of both sexes, with age 18–90 years, and BMI ≥ 30 kg/m^2^ and body composition performed by bioelectric impedance analysis (BIA) and handgrip strength (HS) assessment were collected in a dedicated database. We excluded the data of patients with: (i) missing data for the muscular mass and HS test, and (ii) hydration > 80% [calculated as the percentage of ratio between total body water (L) and fat free mass (kg)] to avoid bias in skeletal muscle mass assessment. When a patient had more than one hospitalization in the time-period considered, we collected data of the first one only.

### 2.2. Data Collection

Patient data were collected upon admission including: (a) demographic and anthropometrics parameters (i.e., age, sex, body weight, height, BMI), (b) co-morbidities (i.e., type and number of concomitant diseases), (c) body composition data obtained by bioelectric impedance analysis, (d) muscle strength assessment by the HS test, and (e) quality of life scores by the Psychological General Well-Being Index (PGWBI) questionnaire. Within 72 h of admission, the bioelectric impedance analysis (BIA), HS test, and the Psychological General Well-Being Index (PGWBI) questionnaire for quality of Life (QoL) were measured.

### 2.3. Anthropometric Parameters

Body weight (kg) and body height (m) were measured to the nearest 0.1 kg and 0.5 cm, respectively, using a mechanical column scale (Scale-Tronix, Wheaton, IL, USA) and a stadiometer (Scale-Tronix, Wheaton, IL, USA); BMI was calculated as body weight in kilograms/height expressed as squared meters (kg/m^2^). 

### 2.4. Body Composition

Body composition analysis was carried out with impedance measurements performed in the early morning after a 12-h overnight fast using a phase-sensitive, single-frequency bioimpedance analyzer (BIA 101, Akern, Pisa, Italy) that applied an alternating current of 400 microÅ at 50 kHz. Before the measurement was taken, each subject removed their clothing and metal jewelry and rested supine for five minutes to equilibrate their body fluids. The impedance measurements were made following the manufacturer’s instructions. The mean coefficient of variation was 1% for within-day and 3% for intra-individual measurements in the steady state condition; 2% was the mean coefficient of variation for the inter-operator variability. 

### 2.5. Skeletal Muscle Mass Index

We applied the following formula by Jansen et al. [11] to calculate the skeletal muscle mass (SMM) in kg, as follows: SMM (kg) = [(Ht^2^/ 0.401 * R) + (3.825 * gender) + (age*- 0.071)] + 5.102, where Ht represents the height (cm) and R represents the BIA resistance measured value. The constants 3.825 and 0.071 were multiplied for the variables sex (male = 1 and female = 0) and age (years), respectively. Finally, we calculated the SMM index (SMMI) as the percentage of the ratio between the SMM (kg) and the total weight in kg [SMMI (%) = (SMM/W) × 100]. 

### 2.6. Muscle Strength

Muscle strength was measured with the HS test using a dynamometer (JAMAR^®^ isometric dynamometer, Cedarburg, WI, USA) on both arms. Three repeated measurements were taken for both the left and right hand, and the mean values were calculated between the measures and registered [12]. The mean difference between the dominant and non-dominant strength was calculated to gauge muscular imbalance, with a difference over 10% indicating neuro muscular imbalance [13].

### 2.7. Psychological General Well-Being Index (PGWBI) Questionnaire

The PGWBI questionnaire [14,15] measures the self-perceived psychological health and general well-being, and intends to assess health-related QoL, or otherwise, to reflect a sense of well-being or distress that includes positive and negative intrapersonal affective or emotional states [15]. The PGWBI questionnaire consists of 22 self-administered items rated on a 6-point Likert scale that range from 0 (= most negative option) to 5 (= most positive option) [15]. The questionnaire includes six non-overlapping dimensions: anxiety, depressed mood, positive well-being, self-control, general health, and vitality [15]. The anxiety dimension evaluates feelings of nervousness, tension, anxiety, worry, and stress. Depressed mood evaluates feelings of depression, hopelessness, and sadness. Positive well-being measures overall happiness and satisfaction with life. Self-control assesses emotional stability and fear of losing control. General health evaluates pain, illness, and ability to perform tasks. Vitality measures energy levels and feelings of vigor or fatigue. The sum of values obtained in each dimension (for a maximum score = 110) determines the overall PGWBI score. The Italian version validated by Grossi et al. [15] had a good internal consistency of the measure, with α values of subscales ranging from 61 to 85. The PGWBI scoring for the QoL evaluation was considered as follows: “good” with a score > 70; “normal” with a score in between 60–70; and “poor” with a score < 60 [15]. 

### 2.8. Criteria for SO Definition

The ESPEN/EASO-SO consensus criteria [9,10] were applied to consecutively satisfy the following diagnostic criteria: (1) muscle strength deficit assessed by HS (<27 kg in men and <16 kg in women), and (2) SMMI deficit (<37% in men and <27.6% in women). Consistent with the study population, the chosen cut-offs were specific for Caucasians. All analyses including screening, diagnosis, and staging in the diagnosis of SO were performed by physicians.

### 2.9. Staging of SO

After the diagnosis of SO was confirmed, a two-step staging evaluation was performed. The staging of SO was determined following the ESPEN/EASO-SO consensus criteria [9,10].

### 2.10. Statistical Analysis 

Results were reported as the means (and standard deviations, SD) for the parametric data, medians and 25th to 75th percentiles (interquartile ranges, IQRs) for nonparametric data, and numbers (%) for categorical data. The bivariate analysis was based on the presence or absence of sarcopenic obesity and divided into groups. We calculated the prevalence of SO and its 95% confidence intervals (95% CI) using the Clopper–Pearson method [16]. The prevalence was calculated for the whole cohort and for each class of age (18–50 years, 51–70 years, and >70 years), BMI range (30–40 kg/m^2^ and >40 kg/m^2^), and sex (women/men). Moreover, we tested the difference among sex or BMI class-specific SO prevalence and age class-specific SO prevalence or using the χ^2^ test (or Fisher test when necessary), or its version of trend, respectively. All analyses were performed using SAS version 9.4 software (SAS^®^ Institute, Cary, NC, USA). Statistical significance was set at the 0.05 level. All *p*-values were two-sided.

## 3. Results

**Figure 1:**
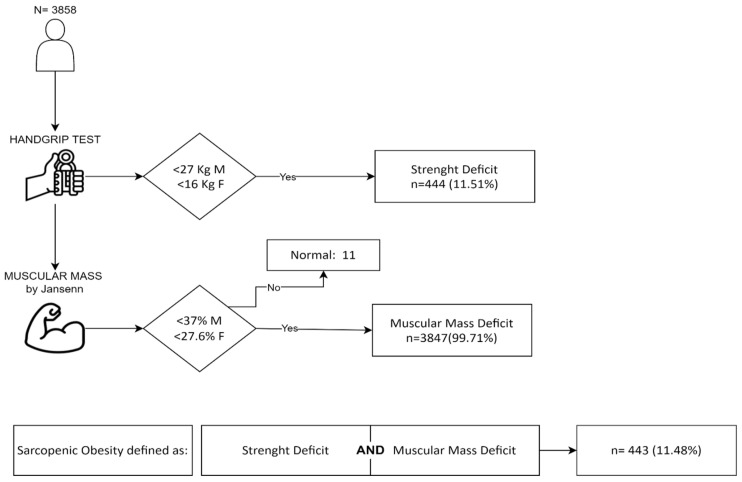
Flowchart of ESPEN/EASO-SO diagnostic criteria application in the whole cohort with the corresponding number and percentage of patients satisfying the diagnostic criteria [9,10].

A total of 6259 patients with a BMI ≥ 30 kg/m^2^ and age between 18 and 90 years were considered. Subsequently, 1647 patients were excluded due to hyper-hydration (total body water > 80% at BIA) and 754 for missing data. The final cohort included 3858 patients (Figure 1). As shown in Table 1, the cohort was mostly composed of women (61%), patients aged between 51 and 70 years (54%), and a mean BMI > 40 kg/m^2^ (59%). About 90% of patients had at least one co-morbidity. Type 2 diabetes, cardiovascular, OSAS, and osteoarticular disease were the most frequent (29%, 33%, 33%, and 41%, respectively) while varices, lymphedema, and BPCO were less prevalent (1% and 4%, respectively) (Table 1). The PGWBI questionnaire data were available for 3136 patients, and 60% of patients had ‘normal’ or ‘good’ QoL scores. 

The flowchart of the ESPEN/EASO-SO diagnostic criteria application with the corresponding patient number is reported in Figure 1. The large majority of the patients (99.71%) had values of SMMI below the considered cut-offs, while 444 patients had a concomitant HS deficit, where the prevalence of SO was 11.48% (95% CI 10.49–12.53%). 

**Figure 2:**
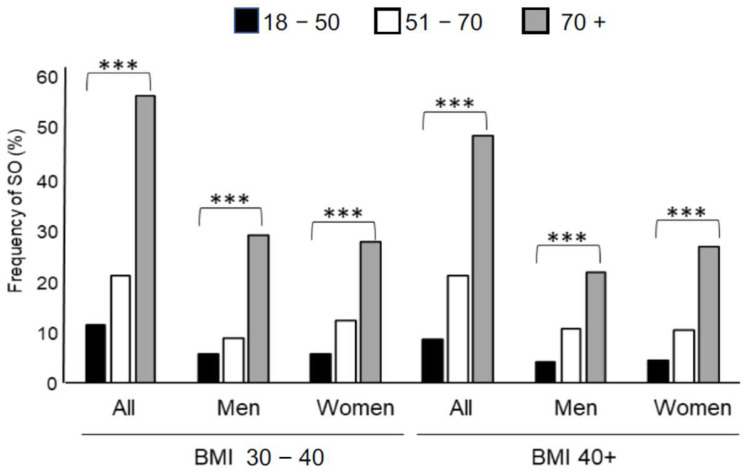
Sarcopenic obesity frequency (%) in all patients with obesity (All) and by sex (men and women, respectively) considering two BMI ranges (30–40 kg/m^2^) and ≥40 kg/m^2^) and by three age classes (18–50 years, black bars; 51–70 years, white bars; ≥70 years, grey bars). The significant trend (*p* < 0.0001) with age is indicated (***).

This prevalence was significantly higher in women (*p* < 0.0001), increasing with age (>70 years, *p* < 0.0001) but not with BMI (*p* = 0.1710). A significant increasing trend was observed with the worsening of QoL scores (*p* < 0.0001) (Table 2). Moreover, we estimated the SO prevalence for different age classes, fixing sex and BMI range (Table 3), observing an increasing significant SO prevalence with age (*p* < 0.0001) (Figure 2). When considering different BMI classes (Supplementary Table S1) and the two sexes (Supplementary Table S2), we did not observe a significant SO prevalence.

We then classified patients for staging, according to the ESPEN/EASO-SO diagnostic criteria [9,10]. Staging I was observed in 21 patients, while staging II was present in 422. The more frequent co-morbidities in SO patients with staging II were osteoarticular degenerative diseases (57%), hypertension/heart failure (38%), type 2 diabetes mellitus (T2DM) (34%), and obstructive sleep apnea syndrome (OSAS) (32%) (Table 4). The age was >70 years in 40% of the SO patients.

## 4. Discussion

When we applied the ESPEN-EASO consensus operational definition of sarcopenic obesity [9,10] with the clinical complications and quality of life (QoL) scores among a large cohort of hospitalized patients with severe obesity, a SO prevalence of 11.48% was observed. The SO prevalence was higher among women with obesity and in patients reporting lower QoL scores. Osteoarticular degenerative disease emerged as the most common clinical complication in the staging of sarcopenic obesity.

When sarcopenia and obesity occur separately, they both compromise health. However, when they coexist, the combination of low muscle mass/functionality and obesity significantly worsens health. This condition leads to heightened disability and cardiovascular risks. The diagnosis of SO is therefore fundamental, especially in a hospital care setting for the management of complications as well as for rehabilitation processes aimed at reducing body weight [6]. In the literature, the SO prevalence values ranged from 6 to 12% in large studies [17], or when considering sex, the range was 11.5% in men and 21% in women with obesity, respectively [17,18]. When considering age, the SO prevalence varied from 2% (among 60–69 years of age) to about 10% (in elderly patients, with age > 80 years) [18,19,20]. Accurate estimation of the prevalence of SO is limited, due not only to the lack of a universally adopted definition of sarcopenia, but also for the use of different body composition assessment techniques. In any case, the frequency of SO established with the ESPEN/EASO diagnostic criteria in the present study is in line with the previously published results [17,18,19,20].

In our dataset, we did not find a significant correlation between BMI and the prevalence of sarcopenic obesity (SO). Interestingly, we observed a higher frequency of SO in individuals with a BMI ranging from 30 to 40 kg/m^2^ compared to those with a BMI ≥ 40 kg/m^2^. It is important to report that fat infiltration in muscles can affect muscle strength independently of BMI [3,4]. Additionally, many patients in the class I and II obesity range (BMI 30–40 kg/m^2^) in our cohort presented metabolic diseases, likely leading to chronic inflammation, insulin resistance, adipokine dysregulation, and increased production of reactive oxygen species (ROS) [21]. These factors can contribute to a decline in muscle quality [22], and as a consequence, reduced muscular functionality [23,24]. The influence of BMI appears to be more closely associated with diseases commonly linked to SO, given that a significant portion of patients had one or more concurrent conditions and functional limitations, notably osteoarticular disease and diabetes. These findings highlight the necessity of implementing a staging system for patients with SO. Consistent with the definition of sarcopenia in the general population [25], age emerged as the primary factor influencing the onset of SO, even among patients with obesity. Previous studies have reported that sarcopenia is positively related to age [25] and will accelerate after 50 years of age. The prevalence varies from 5% to 13% in people in their 70s and increases to 11–50% in people over the age of 80 years [26]. The decline in “muscle quality” (strength-to-mass ratio) with age is a complex study based on the physiological function of SMM that was divided in four domains: force production and transmission, metabolism, thermoregulation, and myokine production and signaling [27]. As suggested by some authors, aging leads to a gradual decline in muscle mass, coupled with an increase in fat mass and alterations in the skeletal mass-to-fat ratio [3,28,29]. This process is further compounded by the detrimental effects of the development of intramuscular adipose tissue (IMAT), chronic inflammation, adipokine dysregulation, and insulin resistance [30,31]. 

Within the examined cohort, approximately 4–6% of diagnosed cases of SO were found in young individuals aged 18–30 years, and about 8–12% in young adults spanning 18–50 years, regardless of their BMI range. This early onset warrants further investigation, particularly in the context of establishing tailored rehabilitation approaches for high risk patients as early skeletal muscle impairment could have a very deleterious effect on health and worsen clinical complications [32].

The sexual dimorphism in human body composition, where adult men typically possess greater total lean mass and lower fat mass compared to women, warrants consideration [29]. Men maintain their lean mass until around their fifth decade of life, after which they experience a physiological slow decline in muscle mass. Women undergo a similar decline in lean mass, but additionally demonstrate a more pronounced increase in fat mass, particularly at menopause age. Consequently, our findings suggest a higher prevalence of sarcopenic obesity (SO) among women, implying that women with obesity may encounter a greater risk of SO compared to men.

Within the examined cohort, the prevalence of SO using ESPEN/EASO diagnostic criteria was predominantly influenced by the presence of functional deficits (HS) rather than the SMM deficit. In fact, although the vast majority of the population exhibited a deficit in muscle mass (99.71%), only 11.48% displayed a concurrent functional deficit in the handgrip strength (HS) test. The high frequency of muscle mass deficit observed may be due to the fact that the studied cohort had an extremely high average BMI (59% of the sample had a BMI > 40 kg/m^2^, Table 1). This indicates the pivotal role of the functional tests as the primary discriminator during the diagnostic process in patients affected by severe obesity. The use of other functional tests (such as the Time Up and Go and/or the six-minute walking test) instead or coupled with HS remains to be explored for SO diagnosis.

The simultaneous occurrence of clinical complications and subsequent disease staging holds significant importance. It is crucial to emphasize that our study cohort consisted of hospitalized individuals participating in rehabilitation programs for various obesity-related disabling conditions, each accompanied by at least one clinical complication. We suggest that these obesity-linked comorbidities may play a role in the development of sarcopenic obesity (SO), likely through distinct yet-to-be-determined mechanisms. Notably, osteoarticular diseases, identified as the most prevalent comorbidity in SO diagnosed patients, play a well-established role in diminishing daily activity and contributing to disability associated with SO [33]. Within Stage II of SO, we observed a significant recurrence of chronic heart failure, type 2 diabetes mellitus (T2DM), and obstructive sleep apnea syndrome (OSAS). As recent reports have highlighted, SO may exert a more substantial influence on metabolic diseases and cardiovascular disease (CVD)-related mortality compared to either sarcopenia or obesity alone [34]. 

Concerning the quality of life among individuals scoring ‘poor’ on the PGWBI (*n* = 1238), 12.6% (*n* = 156 patients) concurrently received a diagnosis of sarcopenic obesity (SO). Recent findings have underscored a heightened QoL impairment in SO patients, a difference that attains clinical relevance when compared to those with obesity alone [35]. Additionally, a noteworthy correlation with a reduced daily step count and a sedentary lifestyle has been reported [36], further emphasizing the impact of SO on daily functioning and well-being. These studies, however, did not used the ESPEN/EASO-SO diagnostic criteria, and these observations remain to be confirmed with dedicated studies to demonstrate whether the QoL score might represent an independent risk for SO onset.

Recently, the SO prevalence, based on the ESPEN and EASO-SO diagnostic criteria, was applied in older Japanese patients undergoing an after-stroke rehabilitation program, where the SO prevalence was 4.5% (5.4% in women and 4.1% in men, respectively), not associated with poor functional outcomes evaluated with the functional independence measure (FIM) [37,38]. Additionally, in a cohort of post-bariatric surgery patients with obesity, a high and variable prevalence of SO (ranging from 7.9 to 23.0%), dependent on the surgery technique used, was reported [39]. Other recent observational studies considered SO prevalence by the ESPEN/EASO diagnostic criteria in patients with obesity, but considered almost exclusively people of geriatric age (>65 years) and in small cohorts of patients [19,20,40]. The data reported here fill a gap in the SO prevalence in geriatric and non-geriatric patients with various obesity classes, especially for BMI ≥ 40 kg/m^2^.

This study presents a possible limitation because our population was not evaluated in real-life conditions, but rather upon admission to a rehabilitation hospital for severe obesity (admission criteria: BMI > 35 kg/m^2^). These results need to be confirmed in different patient cohorts. 

## 5. Conclusions

In conclusion, the application of the ESPEN/EASO-SO diagnostic criteria in a wide cohort of inpatients with severe obesity indicated a prevalence of 11.48% of SO; this prevalence appears to be in line with previous studies and confirms the relevance of age (>70 years) and sex (women), but not of BMI. To conduct a comprehensive clinical assessment of SO, it is imperative to consider the staging (including type and number of concurrent comorbidities) and screening for the quality of life (QoL). Early identification of high-risk patients could facilitate assessing their response to tailored rehabilitation programs or mitigating the onset of disability, thereby enhancing their quality of life.

## Figures and Tables

**Table 1:** Demographic, anthropometric, comorbidities frequencies, and PGWBI scores of the studied cohort.

	*n* (%)
**DEMOGRAPHIC—ANTHROPOMETRIC**	
**SEX**	
Women	2348 (61%)
Men	1510 (39%)
**AGE class (Years)**	
18–50	1142 (30%)
51–70	2090 (54%)
>70	626 (16%)
**BMI class (kg/m^2^)**	
30–40	1595 (41%)
>40	2263 (59%)
**COMORBIDITIES**	
**Endocrine disease**	
T2DM	1101 (29%)
Hypothyroidism	126 (3%)
Pituitary gland disfunction	5 (1%)
Adrenal diseases	0 (0%)
Gonadal diseases	16 (1%)
Dyslipidemia	219 (6%)
Vitamin D deficit	230 (6%)
**Chronic heart diseases**	
Chronic heart failure	1287 (33%)
Varices and lymphedema	55 (1%)
Lower limb ulcers	0 (0%)
**Chronic respiratory diseases**	
OSAS	1284 (33%)
COPD	138 (4%)
**Chronic neurologic and neurodegenerative diseases**	
Peripheral neuropathy	53 (1%)
**Psychiatric disease**	
Depression and psychiatric disease	556 (14%)
**Chronic liver disease**	
Hepatic steatosis	242 (6%)
**Osteoarthritis**	
Osteoarticular degenerative disease	1573 (41%)
**Chronic kidney diseases**	
Kidney failure and stones	93 (2%)
BPH	0 (0%)
**Chronic bowel diseases**	
GERD	5 (1%)
Gastroduodenal ulcers	1 (0%)
Ventral hernia	7 (1%)
Intestinal diverticula	3 (0%)
PGWBI class *	
Good	1258 (40.11%)
Normal	640 (20.41%)
Poor	1238 (39.48%)

Abbreviations: BMI (body mass index), T2DM (diabetes mellitus type 2), OSAS (obstructive sleep apnea syndrome), COPD (chronic obstructive pulmonary disease), BPH (benign prostatic hypertrophy), GERD (gastroesophageal reflux disease). * PGWBI data were available for 3136 patients.

**Table 2:** Sarcopenic obesity prevalence (95% confidence interval, CI) by the ESPEN/EASO-diagnostic criteria.

	*n*	SO	Prevalence (95% CI)	*p*-Value
**OVERALL**				
	3858	443	11.48% (10.49–12.53%)	
**SEX**				<0.0001 ӻ
Women	2348	291	12.39% (11.09–13.79%)	
Men	1510	152	10.07% (8.19–11.21%)	
**AGE (Years)**				<0.0001 ԏ
18–50	1142	53	4.64% (3.50–6.03%)	
51–70	2090	221	10.57% (9.29–11.97%)	
70+	626	169	27.00% (23.55–30.66%)	
**BMI (kg/m^2^)**				0.1710 ӻ
30–40	1595	211	13.23% (11.60–14.99%)	
>40	2263	232	10.25% (9.03–11.58%)	
PGWBI *				<0.0001 ԏ
Good	1258	79	6.28% (5.00–7.77%)	
Normal	640	56	8.75% (6.68–11.21%)	
Poor	1238	156	12.60% (10.80–14.58%)	

* PGWBI data were available for 3136 patients. ӻ: Fisher test. ԏ: Trend test.

**Table 3:** Sarcopenic obesity prevalence (95% confidence interval, CI) by sex, body mass index, and age classes.

Sex	BMI (kg/m^2^)	Age (Years)	No. Total Patients/ Sarcopenic Patients	Prevalence (95% CI)	*p*-Value (Trend Test)
**WOMEN**	30–40	18–50	197/11	5.58% (2.82–9.77%)	<0.0001	
		51–70	502/61	12.15% (9.42–15.33%)		
		70+	213/59	27.70% (21.80–34.23%)		
	>40	18–50	431/19	4.41% (2.67–6.80%)	<0.0001	
		51–70	782/81	10.36% (8.31–12.71%)		
		70+	223/60	26.91% (21.20–33.23%)		
**MEN**	30–40	18–50	157/9	5.73% (2.65–10.60%)	<0.0001	
		51–70	405/36	8.89% (6.30–12.09%)		
		70+	121/35	28.93% (21.05–37.87%)		
	>40	18–50	357/14	3.92% (2.16–6.49%)	<0.0001	
		51–70	401/43	10.72% (7.87–14.17%)		
		70+	69/15	21.74% (12.71–33.31%)		

BMI: body mass index, *p*-value for trend obtained with χ^2^ test or Fisher test.

**Table 4:** Comorbidities in SO patients with staging II.

	*n* (%)
**Endocrine disease**	
T2DM	145 (34%)
Hypothyroidism	11 (3%)
Pituitary gland disfunction	2 (1%)
Adrenal diseases	0 (0%)
Gonadal diseases	0 (0%)
Dyslipidemia	16 (4%)
Vitamin D deficit	17 (4%)
**Chronic heart diseases**	
Chronic heart failure	159 (38%)
Varices and lymphedema	4 (1%)
Lower limb ulcers	0 (0%)
**Chronic respiratory diseases**	
OSAS	137 (32%)
COPD	22 (5%)
**Chronic neurologic and neurodegenerative diseases**	
Peripheral neuropathy	12 (3%)
**Psychiatric disease**	
Depression and psychiatric Disease	58 (14%)
**Chronic liver disease**	
Hepatic steatosis	20 (5%)
**Osteoarthritis**	
Osteoarticular degenerative disease	241 (57%)
**Chronic kidney diseases**	
Kidney failure and stones	16 (4%)
BPH	0 (0%)
**Chronic bowel diseases**	
GERD	0 (0%)
Gastroduodenal ulcers	0 (0%)
Ventral hernia	1 (1%)
Intestinal diverticula	1 (1%)

Abbreviations: T2DM (diabetes mellitus type 2), OSAS (obstructive sleep apnea syndrome), COPD (chronic obstructive pulmonary disease), BPH (benign prostatic hypertrophy), GERD (gastroesophageal reflux disease).

## Data Availability

Data described in the manuscript will be made available upon reasonable request.

## Supplementary Materials

The following supporting information can be downloaded at: https://www.mdpi.com/article/10.3390/jcm13102880/s1: Table S1: Sarcopenic obesity prevalence (95% confidence interval, CI) by sex, age, and body mass index classes; Table S2: Sarcopenic obesity prevalence (95% confidence interval, CI) by age, body mass index classes, and sex.
