# Supplementary material for: Sarcopenia Prevalence among Hospitalized Patients with Severe Obesity: An Observational Study
Source: J Clin Med. 2024 May 13;13(10):2880. doi: 10.3390/jcm13102880 (PMC11122386; doi:10.3390/jcm13102880)
Supplement: Supplementary file 1 [file jcm-13-02880-s001.zip › jcm-2993135-supplementary.pdf]

Supplementary TABLE S1- Sarcopenic Obesity prevalence (95% confidence interval, CI) by sex, age and Body Mass Index classes.

Sex	Age (years)	BMI (kg/m²)	N. total patients/sarcopenic patients	Prevalence (95% CI)	p-value (Fisher test)
Women	18-50	30-40	197/11	5.58% (2.82% - 9.77%)	0.5475
		40+	431/19	4.41% (2.67% - 6.80%)	
	51-70	30-40	502/61	12.15% (9.42% - 15.33%)	0.3175
		40+	782/81	10.36% (8.31% - 12.71%)	
	70+	30-40	213/59	27.70% (21.80% - 34.23%)	0.9144
		40+	223/60	26.91% (21.20% - 33.23%)	
Men	18-50	30-40	157/9	5.73% (2.65% - 10.60%)	0.3613
		40+	357/14	3.92% (2.16% - 6.49%)	
	51-70	30-40	405/36	8.89% (6.30% - 12.09%)	0.4083
		40+	401/43	10.72% (7.87% - 14.17%)	
	70+	30-40	121/35	28.93% (21.05% - 37.87%)	0.3082
		40+	69/15	21.74% (12.71% - 33.31%)	

Supplementary TABLE S2- Sarcopenic Obesity prevalence (95% confidence interval, CI) by age, Body Mass Index classes and sex

Age (years)	BMI(kg/m²)	Sex	N. total patients/sarcopenic patients	Prevalence (95% CI)	p-value (Fisher test)
18-50	30-40	Women	197/11	5.58% (2.82% - 9.77%)	1.0000
		Men	157/9	5.73% (2.65% - 10.60%)	
	40+	Women	431/19	4.41% (2.67% - 6.80%)	0.8586
		Men	357/14	3.92% (2.16% - 6.49%)	
51-70	30-40	Women	502/61	12.15% (9.42% - 15.33%)	0.1303
		Men	405/36	8.89% (6.30% - 12.09%)	
	40+	Women	782/81	10.36% (8.31% - 12.71%)	0.8416
		Men	401/43	10.72% (7.87% - 14.17%)	
70+	30-40	Women	213/59	27.70% (21.80% - 34.23%)	0.8018
		Men	121/35	28.93% (21.05% - 37.87%)	
	40+	Women	223/60	26.91% (21.20% - 33.23%)	0.4338
		Men	69/15	21.74% (12.71% - 33.31%)	
